# Patient Insights Into the Design of Technology to Support a Strengths-Based Approach to Health Care

**DOI:** 10.2196/resprot.5906

**Published:** 2016-08-24

**Authors:** Jelena Mirkovic, Olöf Birna Kristjansdottir, Una Stenberg, Tonje Krogseth, Kurt C Stange, Cornelia M Ruland

**Affiliations:** ^1^ Centre for Shared Decision Making and Collaborative Care Research Division of Medicine Oslo University Hospital HF Oslo Norway; ^2^ Norwegian National Advisory Unit on Learning and Mastery in Health Oslo University Hospital HF Oslo Norway; ^3^ Department of Family Medicine and Community Health (University Hospitals Case Medical Center) Case Western Reserve University Cleveland, OH United States; ^4^ Faculty of Medicine University of Oslo Oslo Norway

**Keywords:** patient strengths, resilience, patient participation, patient-centered care, patient–provider collaboration, user-computer interface, participatory design, chronic disease, patient requirement

## Abstract

**Background:**

An increasing number of research studies in the psychological and biobehavioral sciences support incorporating patients’ personal strengths into illness management as a way to empower and activate the patients, thus improving their health and well-being. However, lack of attention to patients’ personal strengths is still reported in patient–provider communication. Information technology (IT) has great potential to support strengths-based patient–provider communication and collaboration, but knowledge about the users’ requirements and preferences is inadequate.

**Objective:**

This study explored the aspirations and requirements of patients with chronic conditions concerning IT tools that could help increase their awareness of their own personal strengths and resources, and support discussion of these assets in consultations with health care providers.

**Methods:**

We included patients with different chronic conditions (chronic pain, morbid obesity, and chronic obstructive pulmonary disease) and used various participatory research methods to gain insight into the participants’ needs, values, and opinions, and the contexts in which they felt strengths-based IT tools could be used.

**Results:**

Participants were positive toward using technology to support them in identifying and discussing their personal strengths in clinical consultation, but also underlined the importance of fitting it to their specific requirements and the right contexts of use. Participants recommended that technology be designed for use in preconsultation settings (eg, at home) and felt that it should support them in both identifying strengths and in finding out new ways how strengths can be used to attain personal health-related goals. Participants advocated use of technology to support advance preparation for consultations and empower them to take a more active role. IT tools were suggested to be potentially useful in specific contexts, including individual or group consultations with health care providers (physician, nurse, specialist, care team) in clinical consultations but also outside health care settings (eg, as a part of a self-management program). Participants’ requirements for functionality and design include, among others: providing examples of strengths reported by other patients with chronic conditions, along with an option to extend the list with personal examples; giving an option to briefly summarize health-related history; using intuitive, easy-to-use but also engaging user interface design. Additionally, the findings are exemplified with a description of a low-fidelity paper prototype of a strengths-based tool, developed with participants in this study.

**Conclusions:**

Users requirements for IT support of a strengths-based approach to health care appear feasible. The presented findings reflect patients’ values and lists potential contexts where they feel that technology could facilitate meaningful patient–provider communication that focuses not just on symptoms and problems, but also takes into account patients’ strengths and resources. The findings can be used to inform further development of IT tools for use in clinical consultations.

## Introduction

Life with chronic conditions is often very demanding, and requires patients not only to manage different symptoms, problems, and complex treatments (eg, taking medications, adhering to difficult life style adjustments, dealing with emotional consequences such as fear, frustration, and depression), but also to engage various resources (psychological, social, spiritual) [1]. Research shows that to successfully manage chronic illness, patients require support both to learn about and manage their symptoms and problems, and to activate their resources and find new ways to live the best possible life with a chronic illness [2-5]. However, in clinical consultations, health care providers tend to focus on patients’ symptoms, disease diagnoses, and biomedical treatments, without bringing up the topic of patients’ strengths and resources that are vital to making the lifestyle changes needed to manage ongoing chronic illness [6,7]. New ways are needed to support patients as active agents in patient–provider collaboration, to help them discuss both their symptoms and issues but also their resources and strengths, empowering them to develop and adopt new personalized and meaningful self-management plans and strategies.

The term “personal strengths” originates from the field of positive psychology, a discipline that emphasizes health and well-being more than dysfunction and problems [8]. Personal strengths have been defined as the characteristics people use to achieve well-being and to flourish, and include attributes such as hope, gratitude, love of learning, honesty, and humor [9]. Various Web-based strengths inventories can be found to help people identify their personal strengths in workplace and academic settings (eg *,* Values in Action Survey of Character Strengths [10], Strengths Finder 2.0 [11]). The mere act of filling out this type of survey and being aware of one’s strengths can be a helpful intervention [12-14]. However, researchers have suggested that providing some type of support and guidance on how to better use one’s personal strengths through coaching [15], development programs [11], or counseling [16], for example, is even more beneficial.

Although the concept originated in psychology, studies have also explored the use of personal strengths in chronic illness management. Rotegård and colleagues [7] identified a rich repertoire of internal and external strength qualities that cancer patients mobilized to meet their daily living challenges (eg, good mood, optimism, will power, and trust in health care provider). Similarly, Sturgeon and Zautra [17] show that people with chronic pain use various traits and mechanisms to maintain a good life despite their condition (eg, positive emotions, optimism, purpose in life, pain acceptance, active coping, and social engagement). Research suggests that the use of personal strengths attributes has positive effect on health behaviors and outcomes. For example, positive emotions are related to higher patient activation [18] and increased creativity, problem-solving ability, and openness to new experiences and information [19]; resilience is positively related to developing and implementing adaptive coping strategies among patients with chronic pain [17].

To enable easier identification and mobilization of personal strengths, various projects in the areas of psychology, social work, and mental health care have investigated how personal strengths can be identified and used to promote health and well-being. For example, standardized strengths-based assessment tools (both questionnaires and interview guides) have been developed for children and youth with mental health problems [20,21]. Monsen and colleagues [22] propose using a standardized terminology (Omaha System) to describe the strengths of older adults with chronic illnesses and aid development of a whole-person assessment tool. Additionally, various theoretical and practical instruments and guides have been developed for assessment, mobilization, and development of personal strengths in different patient groups (eg, frail elderly persons [23], psychiatric patients [24], and young adults with disabilities [25]).

Despite these guidelines and related work, patients’ personal strengths and resources still often seem to be overlooked in clinical consultations. In a recent study of cancer care, patients reported that clinicians seemed unaware of, did not ask about, discuss, or build on their strengths, which the patients expressed might have helped them become more aware of and better use their potential to improve well-being [7]. A similar finding was outlined by McCammon [26], who concluded that even though strengths-based planning was presented as one of the guiding principles of the care system for children and youth with emotional challenges, and the care teams often require child and family to list their strengths, the care plans frequently neglect to incorporate these strengths into strategies and interventions. These obstacles may be partially related to the dominant paradigm of problem-focused care, as well as difficulty in verbalizing one’s personal strengths [27]. Furthermore, strengths are highly context-specific [28,29], making standardized instruments less meaningful. Computer tailoring, allowing patients to branch into those areas of strengths that are personally relevant, might be a promising approach to support patients, and care providers in eliciting and using patients’ strengths in consultations.

Therefore, in our study we employed participatory methods to explore the requirements and perspectives of patients living with chronic illness on how technology could facilitate bringing the topic of personal strengths and resources into clinical consultations. This study is part of a larger research project called “Incorporating Patient-Identified Personal Strengths into Patient Care” that explores the use of patients’ personal strengths in chronic illness management. The overall goals of the project are to explore how to support patients in identifying and leveraging their personal strengths in health management and how the use of strengths may affect patient activation, motivation for positive change, and patient-centered health care outcomes. The study is done in international collaboration between Case Western University, USA and Oslo University Hospital, Norway, with parallel studies on both sites. In this paper, we describe part of the Norwegian arm of the study, where our main objective was to identify patients’ requirements for an information technology (IT) tool that facilitates awareness and communication of personal strengths in consultation settings, as a means to promote more constructive collaboration and development of strengths-based self-management plans and activities. Additionally, in collaboration with patients, we developed a low-fidelity paper prototype of an IT tool that meet their requirements and can fit into different potential contexts of use.

## Methods

### Study Design

This study uses a rigorous, iterative, qualitative participatory approach to garner the insights and requirements of key end-users of technology to bring patient strengths into health care. The study was conducted between January 2014 and August 2015, and had 3 main phases with the following overall goals:

1. Identify the strengths of people living with chronic illness;

2. Explore patients’ requirements on how technology could be used to promote awareness and discussion of patients’ strengths in consultation settings and grouping strengths into meaningful categories; and

3. Develop a low-fidelity paper prototype for a strengths-based IT-tool in close collaboration with patients, and verify strength category labels.

The goals and methods used in each phase are presented in Table 1.

**Table 1:** Overview of the design process, phases’ aims and methods used.

	Phase aims	Methods
Phase 1 (N=39)	Identify the strengths of people living with chronic illness	Interviews Focus groups
Phase 2 (N=18)	Explore patients’ requirements on how technology could be used to promote awareness and discussion of patients’ strengths in consultation settings Explore patients’ barriers to using technology Explore possible contexts of use Group strengths into categories that are meaningful to patients	Workshops Card sorting exercise
Phase 3 (N=8)	Develop low-fidelity paper prototypes for a strengths-based IT tool in close collaboration with patients Further explore design and functionality requirements and potential new contexts for use Verify strengths category labels	Low-level prototyping Design scenarios

### Participants and Recruitment

Participants were people with chronic obstructive pulmonary disease, chronic pain, or morbid obesity who received care from primary and specialized health care. They were recruited from 4 outpatient rehabilitation or self-management programs in a specialized health care setting. A purposive sampling procedure was used to select information-rich participants of both genders who had lived with one or several chronic illnesses for an extended period of time.

Inclusion criteria were that the person was (1) diagnosed with one or multiple chronic illnesses, (2) more than 18-years old, (3) able to speak and understand Norwegian, and (4) willing to share his/her experiences of living with chronic health challenges. Clinicians from the 4 specialized departments identified potential participants. Those who met inclusion criteria received a letter with information about the study, and the clinicians collected contact information for those patients expressing interest. A researcher (US) then contacted the patient to schedule interviews, focus groups, and workshops. Additionally, a patient representative (TK) was included as a member of the research team with the aim that patients’ voices were integrated into all discussions and final decisions.

This study was planned and performed in compliance with the principles outlined in the Declaration of Helsinki [30], and was approved by the Regional Committees for Medical and Health Research Ethics in Norway and by the Privacy Protection Committee at Oslo University Hospital.

### Phase 1: Identifying Strengths of People Living With a Chronic Illness

In the first phase of this study, we explored how participants described their strengths on personal and interpersonal levels. We conducted 4 focus groups involving 18 patients, 3 paired interviews, and 15 individual interviews between January 2014 and June 2014. During the interviews and focus groups, participants described their strengths and how they used them in managing their everyday lives with chronic illness. Data were analyzed by 2 members of the research team (US, OBK) using qualitative content analysis [31]. Results from this phase, including the detailed list of strengths shared by participants, are being published elsewhere.

### Phase 2: Identifying Patients’ Requirements and Grouping Personal Strengths in Meaningful Categories

This phase involved 5 workshops, with 18 of the same patients who participated in first phase. The workshops were conducted between October 2014 and March 2015.

The first aim was to explore participants’ requirements and barriers for using technology to enhance their awareness of personal strengths and resources, and support discussion of these assets in clinical consultation. Workshops and focus groups are often used for generation of ideas and quickly flush out users’ impressions about a topic or concept, including their opinions, attitudes, preferences, and initial reactions [32]. We created a set of semistructured questions that addressed the topics of possible contexts of use and facilitators and barriers that might potentially emerge. We used an open-ended question format that allowed participants to state their preferences and raise and discuss issues they regarded as important in the group. Because another prototype of the IT tool for patients with low socioeconomic status had been developed in the meantime as part of the other project arm at Case Western University, we showed it to the participants as part of the last 2 workshops to make discussion more concrete and promote generating more ideas. Participants were asked to share their thoughts about how such tools could be used in their care and consultations and to suggest improvements. Workshop sessions were audio-recorded and transcribed. Sessions were analyzed separately by 2 members of research team (JM, TK) using thematic analysis [33]. Coding discrepancies were discussed until consensus was reached.

The second aim of this phase was to explore how participants would expect the personal strengths to be grouped and labeled. We used a card-sorting exercise, asking participants to group strengths identified in Phase 1 into meaningful categories. Card-sorting is a method that reveals how users expect some content to be organized and provides insights into how they group, sort, and label content [34] *.* We started the exercise with an explanation of the method, after which participants were presented with a stack of 91 cards in random order, each labeled with one strength item. Participants were then asked to sort the cards into groups that they felt belonged together and afterward label each group. During the first 3 workshops, some of the participants noted that the large number of items made the card-sorting task very demanding. Therefore, for the next workshop we decided to group strength items that were very similar and present them as one card. To do this the research group together went through the list of strength items and decided in consensus which items were conceptually so similar that they could be grouped together. For example, the strength items “I always look at the things from different perspectives and choose the positive one” and “I am an optimist” were grouped and presented on 1 card. As a result, the number of cards was reduced from 91 to 70 (57 with 1 item, 10 with 2 similar items, and 2 with 4 similar items). The new card organization was used in the final 2 workshops.

At the end of the second phase, the research group analyzed the results of the card sorting exercise from all workshops by going through the list of all proposed categories and organizing and grouping them based on their name, meaning, and the strength items they contained. All decisions were discussed in the research group and made by all team members in consensus.

### Phase 3: Development of a Low-Fidelity Paper Prototype and Verification of Strengths Category Labels

We used collaborative design workshops and iterative low-fidelity prototyping to identify users’ needs regarding the tool’s design and features. Low-fidelity prototyping is a technique often used to visualize possible tool interfaces that could serve as the common language to support discussion with participants about more concrete ideas and requirements [35]. The research team, together with programmers and designers at our research center developed the first version of the prototype based on participants’ insight and feedback in previous study phases. In addition, a design scenario was created that described one hypothetical situation where the tool might be used. The combination of low-fidelity prototyping and design scenarios enabled us to explore functional and design specifications and further discuss potential contexts of use both in the research group and with participating patients [36].

We organized 5 iterative workshops with 6 participants from previous phases and 1 workshop with 2 new patient representatives. Workshops were conducted between May and August 2015. Participants were first given the printed version of the design scenarios that introduce the tool’s context of use. Next, participants were asked to go through the screenshots of the paper prototype, offer feedback and propose changes for both the prototype and the scenario. Each session was audio recorded and participants’ feedback was summarized separately by 2 members of the research team using thematic analysis (JM, TK) [33]. The results were then merged to achieve concordance. The proposed prototype design changes were then jointly discussed among all research team members until consensus was reached concerning which changes to keep, based on the frequency and fundamentality of the issues raised and their alignment to the purpose of the tool.

In this phase, we also performed a final verification of the strength category labels identified in the previous phase. Participants received the list of proposed names for the 6 strengths categories, and were then asked to select and prioritize 3 names for each category that they considered most appropriate and intuitive. All feedback was subsequently analyzed and final category names were selected based on the labels the participants had given highest priority.

## Results

### Participants

A total of 39 patients participated (28/39 women, 72%). The age ranged from 31 to 71 years (mean 50.3, median 49.0). Eighteen were recruited from treatment for a chronic pain condition, 14 for morbid obesity, and 7 for a chronic pulmonary disease.

### Personal Strengths Patients Use in Self-Management and Their Categorization

Phase 1 of the study revealed that patients use a variety of personal strengths to manage their chronic conditions. The strengths descriptions ranged from personal to interpersonal and from specific to general, and included personal characteristics (eg, being optimistic), health-related behavior (eg, exercising, making time for hobbies), and interpersonal and environmental factors (eg, supportive families and work places).

In analyses of data from the card-sorting exercise, with participants, we identified 6 meaningful categories that articulate how participants perceive and categorize their strengths: (1) relations and support, (2) my sources of energy, (3) knowledge about my health, (4) activity and rest, (5) emotions and self-awareness, and (6) positive thoughts and dispositions (Textbox 1).

**Strengths categories elicited from card sorting exercise with examples.**

Relations and support

- I can ask my family for help
- I receive help from competent health care providers
- I appreciate meeting others in a similar situation; then I do not feel so alone

My sources of energy

- I have a hobby I am passionate about
- I spend time on advancing my skills
- I enjoy the feeling of managing my challenges
- I find joy and motivation by spending time with my children and grandchildren

Knowledge about my health

- I have knowledge about and insight into my condition that make me feel more secure
- I have the resources and knowledge to manage my medications
- I follow the physician’s advice regarding medication

Activity and rest

- I do relaxation exercises
- I have a routine for exercising
- I try to find alternative ways to manage things so I can take part in activities that give me joy

Emotions and self-awareness

- I allow myself to focus on myself (not always prioritizing others)
- I do not accept being judged or talked down to
- I have learned to differentiate between sensible thinking and feelings

Positive thoughts and disposition

- I am an optimist
- I try to look at things as challenges, not problems
- I do not perceive myself as sick even if I am in pain

### Technology as a Facilitator for Integrating Strengths Into Patient–Provider Consultations

Analysis of participants’ feedback in workshops and interviews identified 4 main themes: (1) potential benefits of a strengths-based approach in clinical consultation, (2) potential contexts for introducing new strengths-based IT tools, and recommendations when designing (3) functionality, and (4) user interface and interaction.

#### Potential Benefits of a Strengths-Based Approach in Clinical Consultation

Participants confirmed that the consultations they have today most commonly focus on reporting and addressing problems and symptoms. They noted that they often experience the consultation as demanding and stressful, because they usually feel under pressure to remember and report all relevant problems and difficulties and to ask for all the information they need. As a result, they reported lacking the energy or drive to raise and discuss personal strengths and/or health-promoting factors, and/or constructively collaborate with the health care provider in setting goals and making realistic and personalized plans.

Participants said that widening the focus of the consultation to include patients’ strengths and resources could help them look at their situation from another perspective, and instead of focusing on negative aspects–problems and symptoms–see the bigger picture and the positive things and resources they have in their life. Additionally, they noted that paying more attention to personal strengths and resources would help promote active participation from patients during consultations, support them in holding the focus in the consultation on the issues and topics they perceive as relevant, and on defining and discussing personal plans and goals.

Participants also said that addressing strengths and resources in the consultation could motivate them to be more active in performing self-management activities. Many stated that they would like to use the technology not just to help them to identify and discuss their strengths and resources before and during consultation, but also to support them in using the strengths in self-management activities as part of everyday life (eg, in the form of a strengths-based self-management mobile app).

#### Potential Contexts for Introducing New Strengths-Based IT Tools

##### How and Where

Participants stated that technology should be designed to help them identify and reflect on their strengths in more relaxed settings (eg, at home) where they might also get help and support from family members and/or friends, rather than use technology right before or during consultation. In this manner, technology could play a key role in raising the patients’ awareness of their resources and activating them prior to the consultation, and enabling them to prepare for greater participation and more efficient use of the short consultation time with the health care provider.

To further support building the consultation on the patients’ strengths, participants agreed that the health care provider(s) should also prepare for this conversation, (eg, by reading a summary of reported strengths). In this way, health care providers could obtain a more holistic view of the patient and his/her situation. Some of the participants also expressed that it would be useful to begin consultations with the health care provider briefly commenting and reflecting on their summary of strengths and resources. This would give both parties a common understanding and agreement regarding the patient’s current situation.

##### With Whom

Participants identified different contexts in which using strengths-based IT tools could be useful. One example is in preparing for clinical consultations and regular check-ups with different health care providers (eg, medical specialists, physiotherapists, occupational therapists, dieticians, nurses, and social workers). Additionally, it was proposed that this type of IT tools could be useful in preparation for meetings with a multidisciplinary care team (eg, including a physiotherapist, physician, social worker, psychologist, and care coordinator). The tool might help the entire care team get a better overview of the patient’s situation and personal strengths and resources, which could also facilitate the development of personalized care plans. In addition to the use in consultation with health care providers, the participants underlined that strengths-based IT tools could be integrated as part of self-management courses (organized by both health care provider institutions and municipalities). The technology could be used in individual consultations with a course coordinator (to support identifying and mobilizing patient strengths and resources) and in group settings (to increase reflection and group support).

##### When During the Treatment and Recovery

Participants proposed various time-points in time in the process of treatment and recovery when it would be most appropriate to introduce IT tools to raise patients’ awareness of, and to mobilize their personal strengths both for planning and carrying out self-management activities. Some reported that they found the first period after receiving the diagnosis very demanding and stressful, because they needed to adjust and manage the new life situation and treatments. Discussing their strengths and resources with health care providers in these early phases could be perceived as an extra burden rather than as support. They reported that identifying and mobilizing their strengths and resources would be more appropriate later in the illness trajectory, when they start to have better control of their condition and feel ready to try out new things. Conversely, other participants noted that discussion on strengths and resources should be included from the beginning of the treatment to promote valuable help and support for managing new situations and finding useful activities. Additionally, early introduction of patient’s strengths and resources could help health care providers get to know the new patient, and provide support and guidance that fit his/her specific needs. It was concluded that the appropriate timing for introducing a strengths-based tool and having a conversation focused on patients’ strengths varies, depending on the patient’s support needs and readiness to try new health management approaches.

##### How Often

Rather than having just one specific consultation dedicated to identifying and reflecting on their strengths, the majority of participants expressed the need to do this task multiple times. In this way IT tools can be potentially useful to provide them better insights both into their current strengths, but also how they change and adapt over time.

#### Functionality Requirements

All participants agreed that it would be very useful if the IT tool offered them a predefined list of strength items that they could choose from. They especially appreciated the list of strength items that contained examples from others patients living with chronic illness. However, it was agreed that creating an infinite list of strength items would not be feasible. Rather, the proposed best solution was a tool that outlined some relevant strength items and used them as examples and inspiration for identifying and adding one’s own personal strength items in one’s own words.

Participants agreed that they should be given the possibility of identifying and listing their strengths, but also selecting the ones they considered to be most useful and relevant for managing their health at that time. However, using formal scales and ratings to prioritize strength items was found inappropriate in this specific context. Rather, participants universally recommended “prioritizing” and reflecting on up to 5 strengths they found most helpful in achieving their personal goals. Defining goals (both for the specific consultation and for managing health in general) and linking these goals to the identified strengths and resources was suggested as very useful. In addition to defining health-related goals, the participants agreed that it would be useful if the tool also provided them an option to summarize their illness and health-related background and history, as this would help them to concisely outline the information they would like to communicate to the health care providers during the consultation.

#### Design Requirements

Due to their health conditions, participants reported often having problems concentrating. Also, mainly due to age and/or motor problems, they felt that it might be hard for some users to perform long tasks that require complicated interactions. Therefore, the most prioritized design feature for developing strengths-based IT tools was the intuitive user interface and easy interactions. For example, tapping and using check boxes to select items on the screen were reported to be much easier than using more demanding gestures (eg, drag-and-drop). Participants also reported that the use of vertical and horizontal menus could possibly be misunderstood as a requirement to prioritize some items over the others. Therefore, a better choice would be to use alternatives, such as a circular menu where each menu item is color-coded and presented as an equal part of the circle.

Participants said that the interface and design should be engaging and motivating. Some reported irritation when filling out questionnaires, which were often perceived as too long and boring. Thus, advanced design elements (eg, metaphors, multimedia) were proposed to make the tool more attractive and enjoyable.

### Example: A Prototype of a Strengths-Based IT Tool Developed in This Study

**Figure 1:**
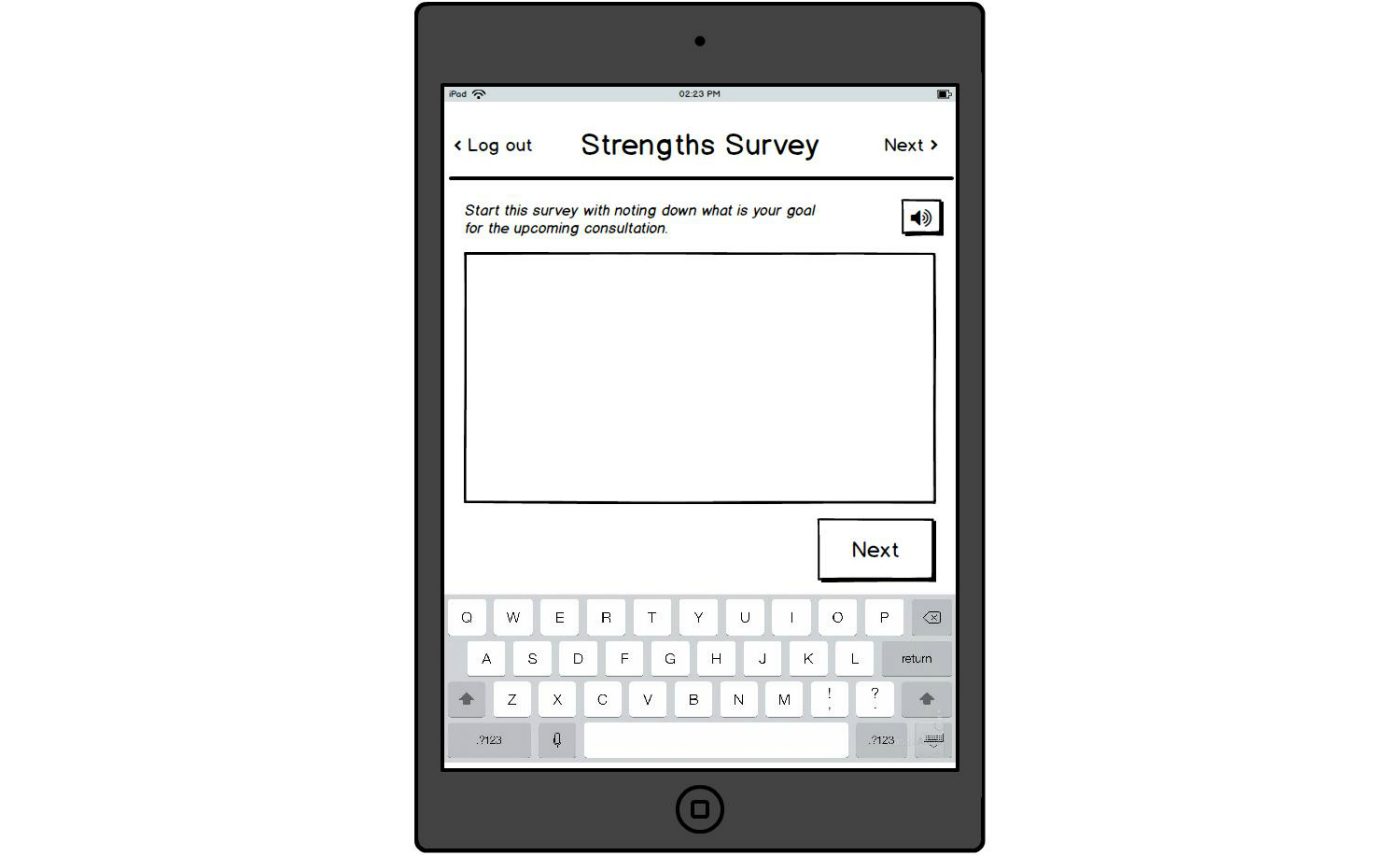
Specifying goal for consultation.

**Figure 2:**
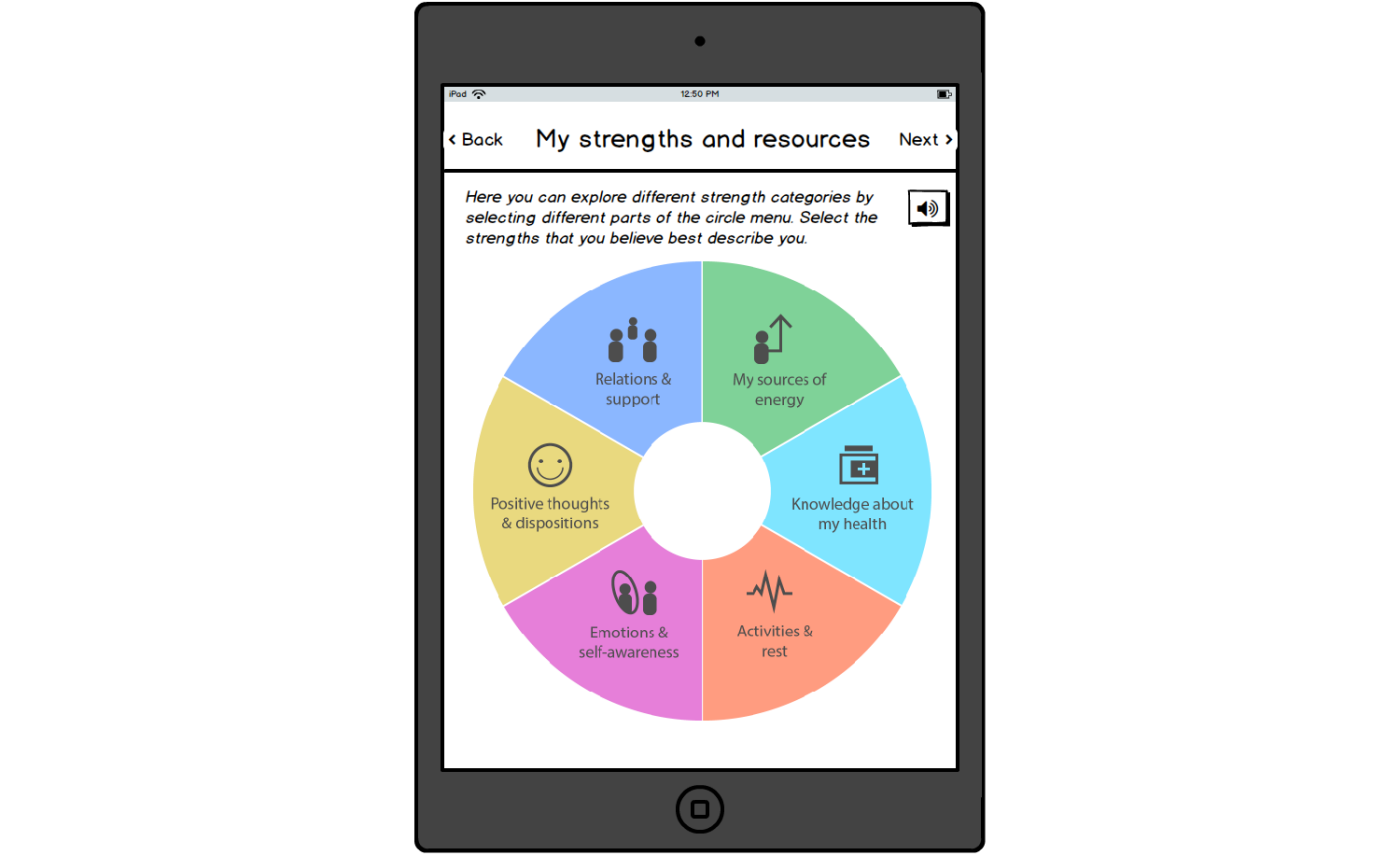
Menu with strength categories.

**Figure 3:**
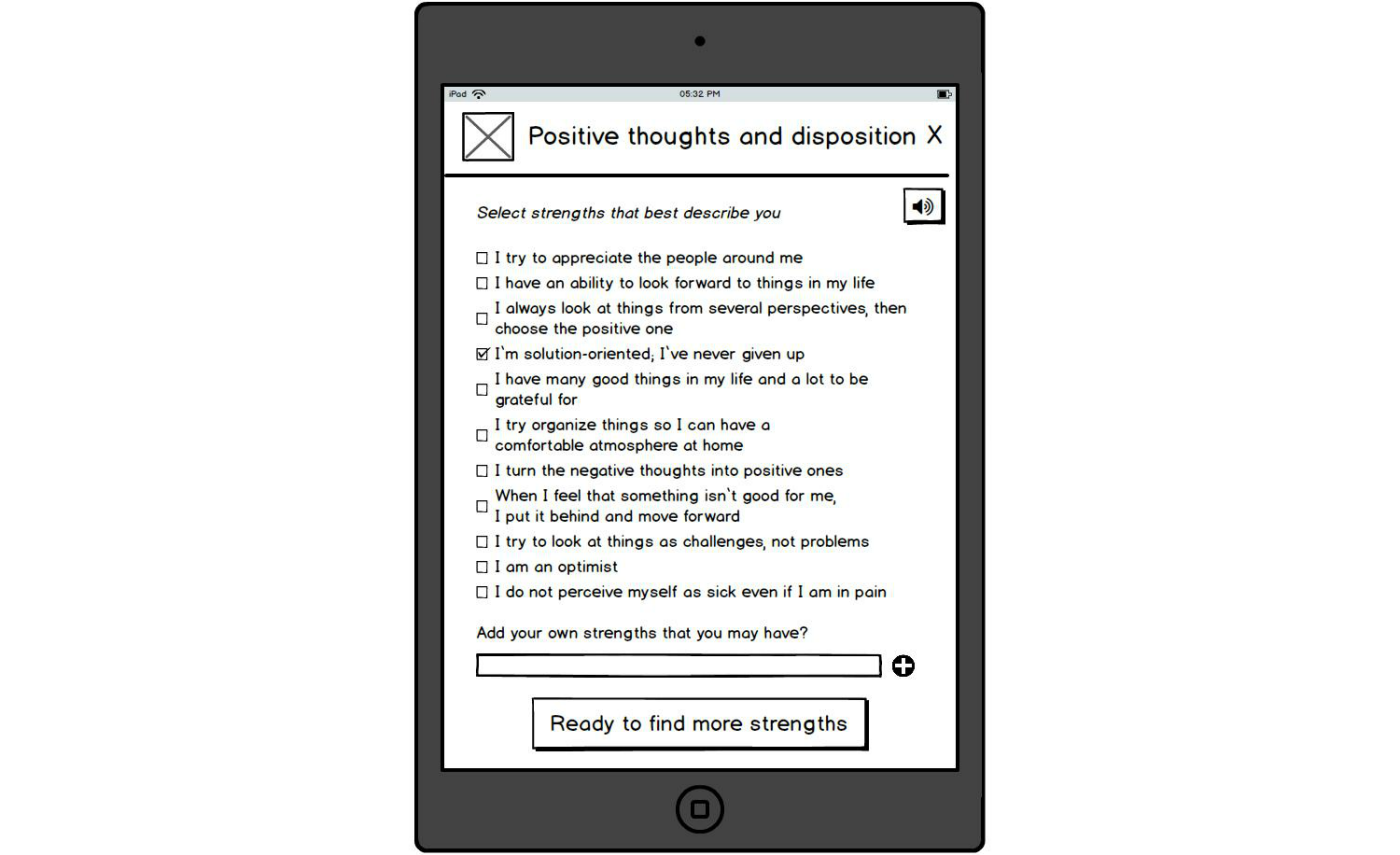
Examples of strengths within one category.

**Figure 4:**
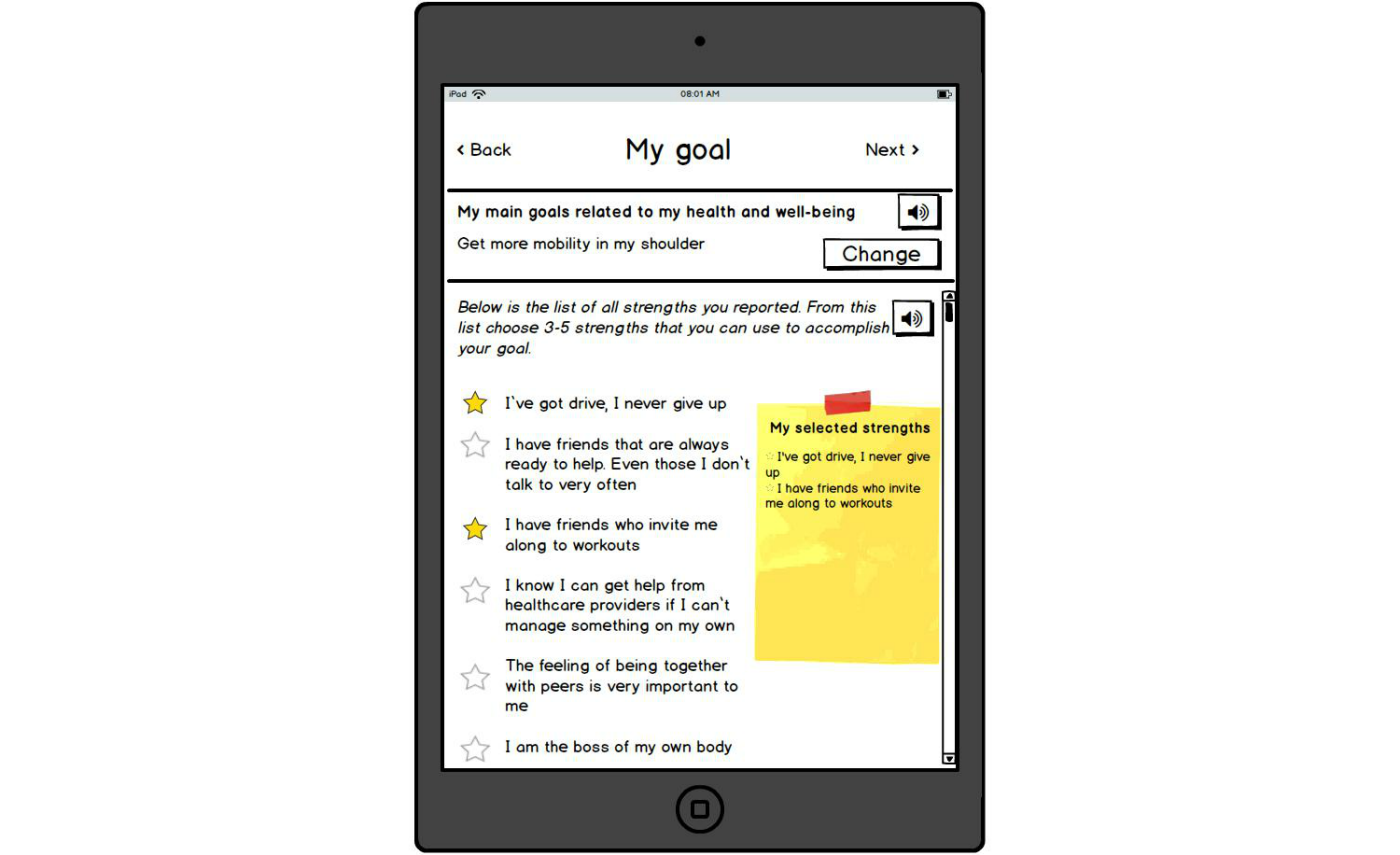
Selection of strengths in relation to a self-defined goal.

**Figure 5:**
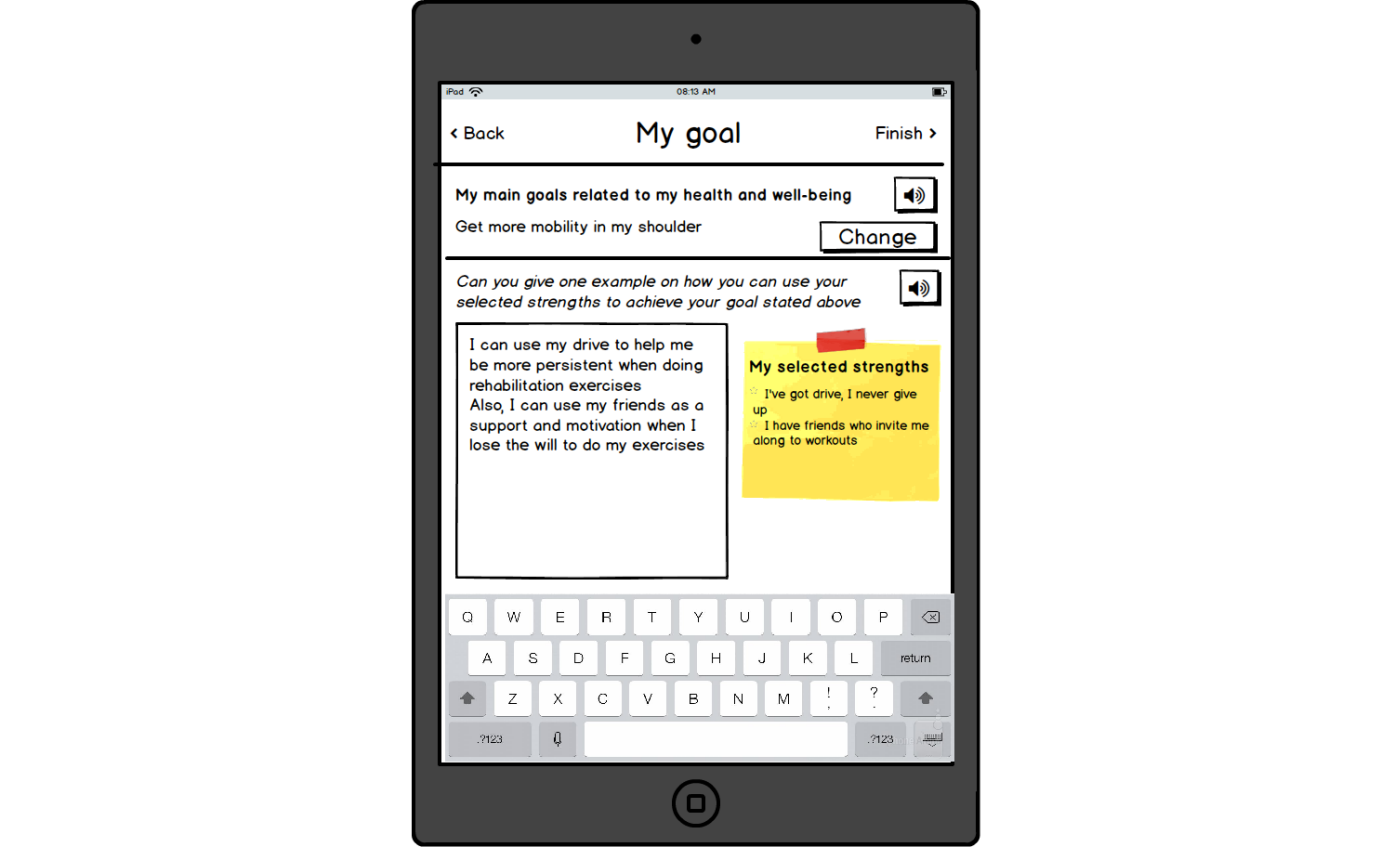
Linking strengths to the health-related goal.

**Figure 6:**
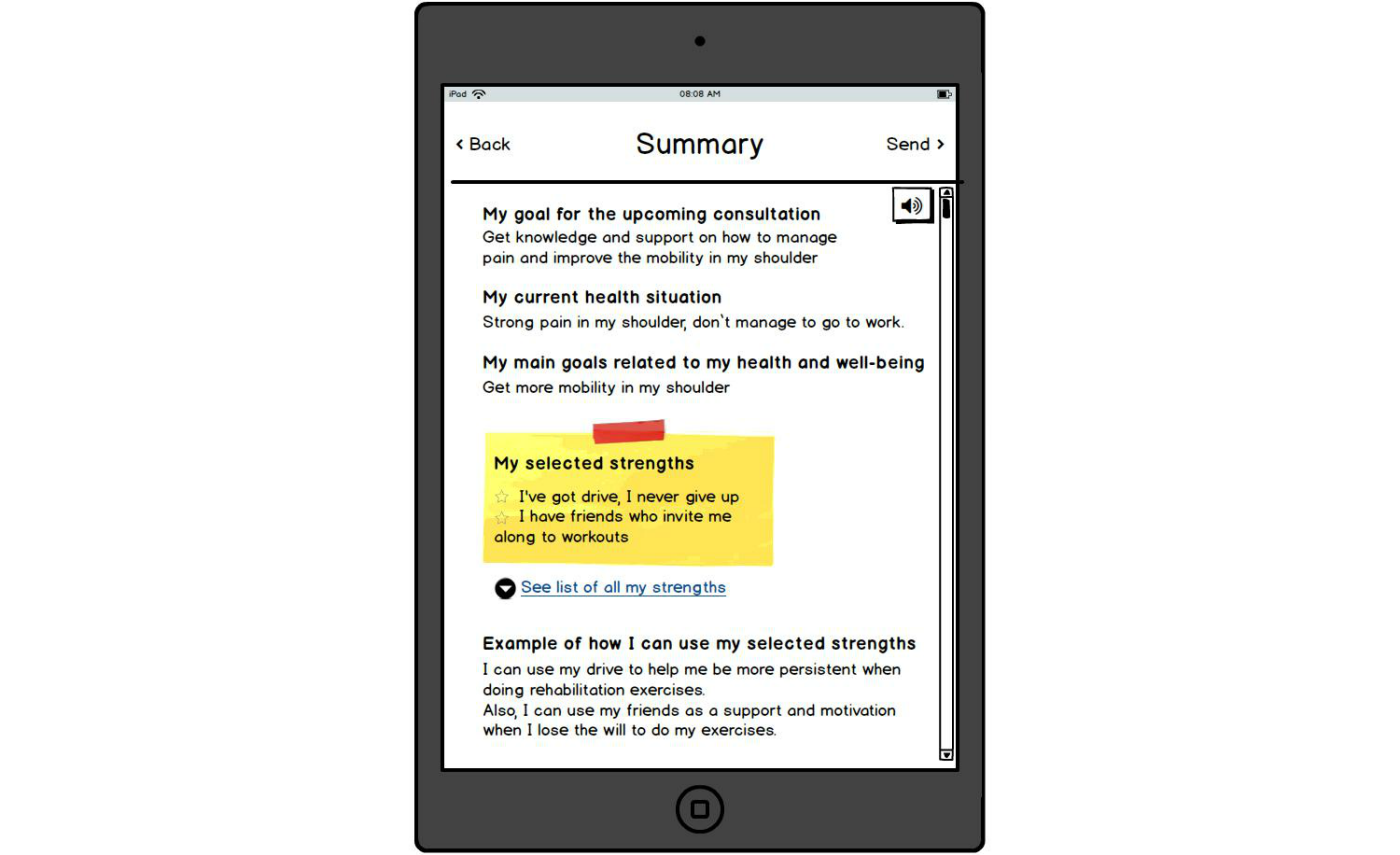
Summary of the strengths assessment.

A low-fidelity paper prototype of a strengths-based IT tool designed in cooperation with participants in the third phase of the study is depicted in screenshots in Figure 1, Figure 2, Figure 3, Figure 4, Figure 5 and Figure 6. The prototype was designed to be applicable to various types of consultation settings that were identified in the study. For illustrative purpose, in Multimedia Appendix 1 we additionally describe a scenario for one potential context of use–preparation for a consultation with a specialist.

## Discussion

### Summary of the Main Findings

Our findings show that patients desire a more holistic health care, and are willing to take a more active role in consultation with health care providers. They also show that patients believe that technology has potential to support them in taking that more active role, by helping them prepare in advance and think about new ways their personal strengths and resources could be used to support self-management activities. Having good, effective communication and collaboration with health care providers is one of the main requirements for successful care for the majority of patients with chronic conditions [1], and various research studies have explored and shown how technology can be used as a facilitator in this process [37,38]. However, most of these studies have mainly emphasized patients’ symptoms and problems, rather than on ways of giving providers a better “whole person” view that integrates, besides symptoms, also patient’s strengths and resources, and that could be used for establishing more personalized and attainable care plans [39,40]. The present study is, to our best knowledge, the first to explore what people with chronic illness consider important in the design of an IT tool to aid assessment of their personal strengths and support them in discussing these in a consultation with a health care provider.

Participants in our study outlined the requirements for a tool that would not just help them identify their personal strengths but also help them reflect and pinpoint the strengths they think could be useful for achieving their own personal health-related goal. This relation between personal strengths and goals has previously been addressed in related research. For example, coaching psychology shows that use of personal strengths to pursue meaningful, personally relevant goals is associated with better progress toward those goals, which is in turn associated with psychological need fulfillment and enhanced well-being [41]. Using technology to support people in linking their personal strengths and health-related goal(s), could therefore be an important first step for making more attainable and personalized self-management plans and choosing activities (on one’s own or in collaboration with the health care provider).

### Strengths and Limitations of the Study

Current trends in the development of health care interventions outline that they have to be designed not only to be useful, acceptable, and nonharmful, but also pleasant and engaging [42,43]. To accomplish this when creating an IT-based tool, it is important to elicit users’ requirements regarding system functionality and usability, identify what creates positive value for individual persons in their own context, obtain meaningful user-experiences based on people’s thoughts and beliefs, and map these to system design (value co-creation) [44]. In this study, we worked closely with patients to elicit their needs, opinions, values, and preferences, and on that basis jointly created the a prototype for a tool that would be both engaging (as it would help patients identify and use their own strengths and resources), and motivating (as it would inspire them to take a more active role in collaborating with health care providers and managing their health). We collected various feedback from users on how to make the tool more engaging, in terms of interface design (eg, use circular menus to make all strength categories look equally important, use of videos to help users understand concepts, and categories of strengths), and related to user experience (eg, giving examples of strengths reported by other patients with chronic conditions to help people relate and reflect on their own situation). Additionally, organizing the strength items into categories that are logical and meaningful to users supports customizable design that enable users to select only categories that are relevant in their context. By inductively identifying the patient perspective, use of qualitative participatory approach and method supported us to set-up future work to design IT support that is relevant to the needs of chronic illness patients for a strengths-based approach to health care.

The main limitation of the work presented in this paper is that it includes the perspectives of just one group of stakeholders–patients. Therefore, subsequent phases of the project will involve a broader group of stakeholders–both the types of health care providers identified as potentially relevant in this study, and additional patients with the same and new diagnoses–to further explore feasibility and possibility for implementation of strengths-based IT tools.

### Implications for Designing IT Support for a Novel Strengths-Based Approach to Health Care

All participants in our study agreed on the importance of a conversation about the patient’s strengths and resources, and how to build on them in self-management of a chronic condition. Previous studies have suggested that although adding a discussion on personal strengths to the consultation may indeed require some extra time, this is assumed to result in better health care, user activation, and promotion of health [13,45]. However, the optimal time point for having this type of conversation remains unclear. Identifying consultation settings in which both the patient and the clinician agree that a new approach is needed, and then bringing patient strengths into these interactions is likely to maximize the receptivity to a new strengths-based approach. Such an approach can be used in both individual and group consultations, as well as outside health care settings (eg, self-management courses). Some previous research in related fields promotes paying attention to patients’ strengths in a consultation setting by using informal qualitative methods, such as proposing an open question about personal strengths (eg, “We cannot only talk about problems. I also want to hear about your strong points. Which of these strong points do you normally use to stay (or become) well?” [46]). In this setting technology could have the great potential to help patients prepare for this conversation by supporting their efforts to select and specify their strengths in advance and potentially even come up with their own ideas about how would they like to use them to manage their health condition. The question of when this kind of dialogue should be initiated, by whom, and which is the best moment to introduce technology as a facilitator in this process remains open, and should be addressed in future research.

### Conclusion

Our study provides initial insights into patients’ requirements for developing new IT tools that help them in identifying and reflecting on their personal strengths and support discussion of these assets in consultations with health care providers. We conclude that technology has great potential to be used to create novel opportunities for activating and empowering patients. Developers and designers of strengths-based IT tools should be aware of these requirements and attempt to accommodate them during design and development of new technologies for use in clinical consultations.

## Appendix

Multimedia Appendix 1 Scenario illustrating potential use of the strengths-based IT tool and associated procedures.

## Abbreviations

IT: information technology

## References

[1] Liddy C, Blazkho V, Mill K (2014). Challenges of self-management when living with multiple chronic conditions: systematic review of the qualitative literature. Can Fam Physician.

[2] Schulman-Green D, Jaser S, Martin F, Alonzo A, Grey M, McCorkle R, Redeker NS, Reynolds N, Whittemore R (2012). Processes of self-management in chronic illness. J Nurs Scholarsh.

[3] Corbin J, Strauss A (1988). Unending Work and Care: Managing Chronic Illness at Home (Jossey Bass Social and Behavioral Science Series).

[4] Lorig KR, Holman H (2003). Self-management education: history, definition, outcomes, and mechanisms. Ann Behav Med.

[5] Zuidema RM, van Gaal BGI, van Dulmen S, Repping-Wuts H, Schoonhoven L (2015). An online tailored self-management program for patients with rheumatoid arthritis: a developmental study. JMIR Res Protoc.

[6] Kruse RL, Olsberg JE, Shigaki CL, Parker Oliver DR, Vetter-Smith MJ, Day TM, LeMaster JW (2013). Communication during patient-provider encounters regarding diabetes self-management. Fam Med.

[7] Rotegård AK, Fagermoen MS, Ruland CM (2012). Cancer patients' experiences of their personal strengths through illness and recovery. Cancer Nurs.

[8] Peterson C, Seligman MEP (2004). Character Strengths and Virtues: A Handbook and Classification.

[9] Seligman M, Csikszentmihalyi M (2000). Positive psychology. An introduction. Am Psychol.

[10] VIA Insititute on Character Take Off and Get to Know Your Character strengths.

[11] Galup Strengths Center.

[12] VIA Insititute on Character (2016). VIA Strengths @ Work.

[13] Flückiger C, Caspar F, Holtforth MG, Willutzki U (2009). Working with patients' strengths: a microprocess approach. Psychother Res.

[14] Asplund J, Lopez S, Hodges T, Harter J (2007). The Clifton StrengthsFinder® 2.0 Technical Report: Development and Validation.

[15] Madden W, Green S, Grant A (2011). A pilot study evaluating strengths-based coaching for primary school students: Enhancing engagement and hope. International Coaching Psychology Review.

[16] Littman-Ovadia H, Lazar-Butbul V, Benjamin BA (2014). Strengths-based career counseling: overview and initial evaluation. Journal of Career Assessment.

[17] Sturgeon JA, Zautra AJ (2010). Resilience: a new paradigm for adaptation to chronic pain. Curr Pain Headache Rep.

[18] Hibbard JH, Mahoney E (2010). Toward a theory of patient and consumer activation. Patient Educ Couns.

[19] Fredrickson BL, Losada MF (2005). Positive affect and the complex dynamics of human flourishing. Am Psychol.

[20] Benson PL, Leffert N, Scales PC, Blyth DA (2012). Beyond the “village” rhetoric: creating healthy communities for children and adolescents. Applied Developmental Science.

[21] Harniss MK, Epstein MH, Ryser G, Pearson N (1999). The behavioral and emotional rating scale: convergent validity. Journal of Psychoeducational Assessment.

[22] Monsen KA, Holland DE, Fung-Houger PW, Vanderboom CE (2014). Seeing the whole person: feasibility of using the Omaha System to describe strengths of older adults with chronic illness. Res Theory Nurs Pract.

[23] Kivnick HQ, Murray SV (2001). Life strengths interview guide: assessing elder clients' strengths. Journal of Gerontological Social Work.

[24] Bellier-Teichmann T, Pomini V (2014). Evolving from clinical to positive psychology: understanding and measuring patients’ strengths: a pilot study. J Contemp Psychother.

[25] Kim JH, Reid CA, McMahon B, Gonzalez R, Lee DH, Keck P (2016). Measuring the virtues and character traits of rehabilitation clients: the adapted inventory of virtues and strengths. J Occup Rehabil.

[26] McCammon SL (2012). Systems of care as asset-building communities: implementing strengths-based planning and positive youth development. Am J Community Psychol.

[27] Blundo R (2001). Learning strengths-based practice: challenging our personal and professional frames. Families in Society: The Journal of Contemporary Social Services.

[28] Biswas-Diener R, Kashdan TB, Minhas G (2011). A dynamic approach to psychological strength development and intervention. The Journal of Positive Psychology.

[29] Niemiec R (2013). VIA Character Strengths: Research and Practice (The First 10 Years).

[30] World Medical Association General Assembly (2001). World Medical Association Declaration of Helsinki. Ethical principles for medical research involving human subjects. Bull World Health Organ.

[31] Graneheim UH, Lundman B (2004). Qualitative content analysis in nursing research: concepts, procedures and measures to achieve trustworthiness. Nurse Educ Today.

[32] Courage C, Baxter K (2005). Understanding Your Users: a Practical Guide to User Requirements: Methods, Tools, and Techniques.

[33] Joffe H, Yardley L (2004). Content and Thematic Analysis. Research Methods for Clinical and Health Psychology.

[34] Spencer D (2009). Card Sorting: Designing Usable Categories.

[35] Snyder C (2003). Paper Prototyping: The Fast and Easy Way to Design and Refine User Interfaces.

[36] Rudd J, Stern K, Isensee S (1996). Low vs. high-fidelity prototyping debate. Interactions.

[37] Heyn L, Ruland CM, Finset A (2012). Effects of an interactive tailored patient assessment tool on eliciting and responding to cancer patients' cues and concerns in clinical consultations with physicians and nurses. Patient Educ Couns.

[38] White A, Danis M (2013). Enhancing patient-centered communication and collaboration by using the electronic health record in the examination room. JAMA.

[39] Robinson JH, Callister LC, Berry JA, Dearing KA (2008). Patient-centered care and adherence: definitions and applications to improve outcomes. J Am Acad Nurse Pract.

[40] Arora NK (2003). Interacting with cancer patients: the significance of physicians' communication behavior. Soc Sci Med.

[41] Linley P, Nielsen K, Gillett R, Biswas-Diener R (2010). Using signature strengths in pursuit of goals: Effects on goal progress, need satisfaction, and well-being, and implications for coaching psychologists. International Coaching Psychology Review.

[42] Sander T (2011). Positive Computing.

[43] Ludden GD, van Rompay TJL, Kelders SM, van Gemert-Pijnen JEWC (2015). How to increase reach and adherence of web-based interventions: a design research viewpoint. J Med Internet Res.

[44] Kirah A (2009). Co-creation: a new way of doing business in an age of uncertainty. Open Source Business Resource.

[45] Hollnagel H, Malterud K (2000). From risk factors to health resources in medical practice. Medicine, Health Care and Philosophy.

[46] Tedeschi R, Kilmer R (2005). Assessing strengths, resilience, and growth to guide clinical interventions. Professional Psychology: Research and Practice.

