# Supplementary material for: Patient Insights Into the Design of Technology to Support a Strengths-Based Approach to Health Care
Source: JMIR Res Protoc. 2016 Aug 24;5(3):e175. doi: 10.2196/resprot.5906 (PMC5014985; doi:10.2196/resprot.5906)
Supplement: Multimedia Appendix 1 [file resprot_v5i3e175_app1.pdf]

Scenario illustrating potential use of the strengths-based IT tool and associated procedures

Peter is 56 years old. He had a serious traffic accident two years ago and since then he has been living with chronic pain in his right shoulder. As a result, he has limited shoulder mobility and flexibility. He has regular follow up consultations with his primary physician and he went to a physiotherapist to get advice and help on how to get better mobility in his shoulder. However, since his situation did not improve over the last year his physician referred him for a consultation with a pain specialist at the local hospital. One week before the consultation he receives a letter from the pain clinic reminding him about the approaching appointment. At the end of the letter he finds information about an available IT tool that might help him prepare for the consultation and give him support to identify and reflect on his own strengths and resources that he could use to manage his current health condition. In addition to the link to the web page where he can access the tool, the text briefly introduces the tool, explains what advantages it might offer him and informs him that it will take approximately 10-15 minutes to use it.

That evening Peter starts thinking about the upcoming consultation and he decides to try out the IT tool that he read about in the letter. He types the web address into the web browser on his iPad. The first page provides an introduction of the tool in the form of text, audio file, and a short animated video. After watching the introductory video, he decides that the tool could be useful for him, so he proceeds to the next page where he is asked to log in using standard strong authentication mechanisms he usually use when managing his private and sensitive information online. Once he has logged in, communication continues over a secure connection, and he is asked to type in his personal goal for the upcoming consultation (Figure 1) and, if he wishes, summarizes his illness history and current health status. When he finishes filling out this part, next screen shows the circular menu with six main strength categories (Figure 2). He chooses “My sources of energy” category and selects a couple of items from the list that he thinks best describe him (Figure 3). He also decides to type in and describe a couple more items that he feels give him energy but that he did not find in the list (Figure 3). Afterwards he goes through a couple of the other categories from the main menu and adds more strengths to his list. When he finishes with identifying and specifying his main strengths he go to the next step, where he is asked to write his overall health-related goal and select up to five strengths from his list that he would like to use to accomplish this goal (Figure 4). Additionally, he is given an option to describe in greater detail how he would like to accomplish this goal and what help and support he would need to succeed in this (Figure 5). When he finishes this step, he gets a preview of all the information he typed and selected in the tool. He decides he would like to share this with the specialist at the clinic and sends the report over secure connection (Figure 6). He also saves the report on his iPad, so he can access it and review it more later.

Before the consultation, the pain specialist at the local hospital reads through the short report Peter submitted, reviewing his current health status, goals and the top

five personal strengths he reported he would like to use. In the consultation, the specialist first hears out Peter’s concerns and symptoms. In addition, they talk about the goal, health issues, and strengths and resources Peter reported in the tool. Based on this common understanding of the Peter’s situation, Peter and the specialist together discuss how Peter’s issues can be best addressed and jointly make plans and choose activities that fit and build upon his resources and strengths.
